# Twisting Steel Wires with a Drill: A New Surgical Technique

**DOI:** 10.1111/os.12457

**Published:** 2019-04-10

**Authors:** Meng‐qiang Fan, Jiang Hua, Jie‐feng Huang

**Affiliations:** ^1^ Department of Orthopaedics The First Affiliated Hospital of Zhejiang Chinese Medical University Hangzhou China; ^2^ The First Clinical College Zhejiang Chinese Medical University Hangzhou China

**Keywords:** Twist, Wires, Electric drill

## Abstract

Stainless steel wires are often used to fix specific types of fractures in orthopaedic surgery using pliers. This article aims to introduce a new technique to twist steel wires by using an electric drill. The steps before twisting the wire are the same as usual. Our technique is as follows. First, tighten the two ends of wire by using pliers, then insert both the ends of wires into the drill and hold in place. Second, set the drill to reverse mode. Third, start turning the drill, then the two wires begin to intertwine and tighten. It is important to stop turning before the wire (the twisted part) begins to bend. Finally, cut the twisted part of wire in place, and bend the wire stump. This technique can achieve a better appearance while saving the strength of surgeons. It has the same clinical effect as the traditional method. This technique provides a new method for surgery and wide clinical application.

## Introduction

Stainless steel wires are often used to fix specific types of fractures in orthopaedic surgery. We usually use stainless steel wire in patellar fractures^1^, olecranon fractures, periprosthetic fractures, Jones fractures^2^, and ulnar coronal avulsion fractures. It is worth mentioning that, for example, in patella fractures, the loosening of internal fixation is not rare among patients suffering from patella osteosynthesis^3^. However, there are problems with using pliers to tighten the wire to fix displaced fracture fragments. First, surgeons may exert uneven force when twisting, leading to an inaesthetic wire shape. Second, the process of twisting wires consumes doctors’ strength.

Our technique can solve these problems by using an electric drill to twist the steel wires. Compared with the traditional method, the technique of twisting wire has uniform force, results in a good appearance, and saves the strength of surgeons.

## Technique

### *Typical Case*

A 34‐year‐old man was admitted to our hospital for treatment 2 hours after a falling injury. During the physical examination by the attending physician, it was evident that the patient was experiencing left knee pain, swelling was obvious, and joint movement was significantly limited. Further examination revealed that the patient's left patella was touching the step‐like fracture line. The patient was considered to have a closed fracture of the left patella.

X‐rays showed a transverse fracture of patella. A computed tomography scan showed a transverse patellar fracture with fracture displacement greater than 2 mm.

Relative contraindications were excluded for open reduction and internal fixation of the left patella.

One week after admission, the swelling of the patient's left knee joint was eliminated, and the patient underwent open reduction and internal fixation. Under general anesthesia, surgery was performed in supine position with the injured knee extended. The tourniquet was used. A vertical central incision was made at the left knee joint and the hematoma tissues were removed from the fracture site.

Antibiotics were routinely used to prevent infection 24 hours after surgery. Functional exercise began after the operation. Stitches were removed 2 weeks after surgery. X‐rays were taken for review at the end of the surgery, and after 4 weeks, 12 weeks, 6 months, 12 months and 24 months. Internal fixation was removed 1 year after surgery. Hospital for special surgery (HSS) score, visual analog scale (VAS), and range of motion (ROM) were used for functional, pain, and activity scores at 4 weeks, 12 weeks, 6 months, 12 months, and 24 months after surgery.

The plain radiography was used to assess union, and the union time was 12 weeks. At last follow‐up, the postoperative result in terms of the HSS score was 96. The final knee joint arc of flexion motion was 133°, with an arc of extension motion of 0° at last follow‐up.

During 2 years of follow‐up, no secondary fracture or loosening of the wire occurred. Postoperatively, there was no knee stiffness, extensor muscle weakness, or traumatic osteoarthritis.

### *Surgical Technique*

This surgical technique of wire‐twisting was approved by the hospital's ethics committee. The steps before twisting the wire are the same as usual. Our technique is as follows. First, tighten the two ends of wire by using pliers, then insert both the two ends of wires into the drill and hold in place. Second, set the drill to reverse mode. Third, start turning the drill; the two wires then begin to intertwine and tighten. It is important to stop turning before the wire (the twisted part) begins to bend. Finally, cut the twist part of wire in place, and bend the wire stump.

**Figure 1:**
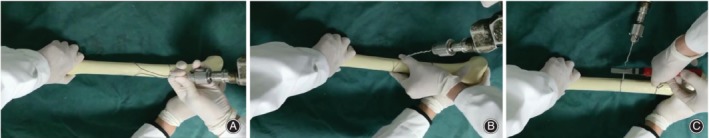
In vitro demonstration. (A) Insert both the two ends of wires into the drill and hold in place. (B) Set the drill to reverse mode. Start turning the drill; then the two wires begin to intertwine and tighten. Stop turning before the twisted part of wire begins to bend. (C) Cut the twisted part of the wire in place and bend the wire stump.

**Figure 2:**
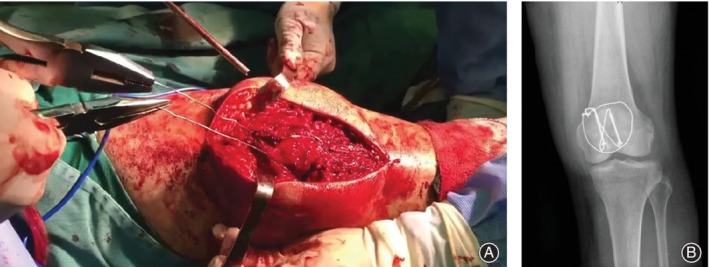
Application in surgery. (A) Application in patellar fracture. During application in surgery, we usually tighten the ends of the wire with a vice first. (B) X‐ray of the first day after operation for patellar fracture.

In vitro demonstration is presented in Fig. 1, and the application in surgery is depicted by Fig. 2.

## Discussion

Our novel method of twisting wires with an electric drill has been used successfully and safely for the past 11 years in our hospital on over 300 patients without wires loosening and breaking after surgery. The surgical technique has the same surgical indication as the traditional method. We use this surgical technique routinely for fixation of fractures, including for patellar fractures, olecranon fractures, periprosthetic fractures, Jones fractures, and ulnar coronal avulsion fractures.

We usually set the drill in reverse mode because the torque of the reverse mode is less than the forward mode, and the speed of the reverse mode is slower than that of the forward mode. The reverse mode is easier for surgeons to control and they can avoid excessive bending or even breaking of the wire (the twisted part) due to too much torque. Furthermore, we do not quantify the time to stop turning but, rather, simply suggest stopping before the twisted part of the wire is just bent. If not, the fracture may be fixed too tightly, and the twisted part of the wire may break.

Our study had a particular limitation. Although the clinical experiment results are good, we have not verified the strength of postoperative steel wire from the perspective of mechanics, which can be verified in clinical practice in the future.

## Supporting information


**Videos S1** http://onlinelibrary.wiley.com/doi/10.1111/os.12457/suppinfo
